# Abemaciclib-associated acute interstitial nephritis successfully treated with glucocorticoids: a case report and literature review

**DOI:** 10.1186/s12882-025-04442-3

**Published:** 2025-09-29

**Authors:** Mari Kumano, Yujiro Maeoka, Yuki Teragawa, Hiroki Yanagidani, Maria Yoshida, Akira Takahashi, Takao Masaki

**Affiliations:** Department of Nephrology, Hiroshima University Hospital, 1-2-3 Kasumi, Minami-ku, Hiroshima, 734-8551 Japan

**Keywords:** Acute interstitial nephritis, Abemaciclib, CDK4/6 inhibitor, ATN, AKI, Glucocorticoid

## Abstract

**Background:**

Drug-induced acute interstitial nephritis (DI-AIN) is the most common type of AIN. DI-AIN occurs when medications trigger a T cell-mediated immune response that promotes tubulitis and interstitial inflammation with eosinophils, often resulting in acute kidney injury (AKI) with nephromegaly. Recently, prolonged use of cyclin-dependent kinase (CDK) 4/6 inhibitors, as oral molecular-targeted drugs for breast cancer, was identified as a cause of AKI, including AIN and acute tubular necrosis (ATN). To date, there have been no reported cases of AIN associated with the use of abemaciclib, a CDK4/6 inhibitor.

**Case presentation:**

A 66-year-old Japanese woman presented with persistent diarrhea and nausea shortly after the initiation of abemaciclib for breast cancer and was subsequently referred to our hospital with severe renal dysfunction (blood urea nitrogen, 128.7 mg/dL; creatinine, 15.16 mg/dL). Based on her elevated renal tubular damage markers and bilateral renal enlargement, acute renal failure was suspected. A renal biopsy revealed interstitial infiltration of mononuclear cells and eosinophils, along with tubulitis and epithelial cell damage within the renal tubules, suggesting AIN caused by abemaciclib. The renal function improved with glucocorticoid therapy following fluid replacement for pre-renal AKI, and the serum creatinine decreased to approximately 2 mg/dL within 2 months.

**Conclusions:**

We report a case of biopsy-proven AIN that developed shortly after the initiation of abemaciclib, leading to severe renal dysfunction with nephromegaly. While prolonged use of CDK4/6 inhibitors can cause both AIN and ATN, AIN can also occur after short-term use, highlighting the importance of a renal biopsy to determine the need for glucocorticoid therapy.

**Clinical trial number:**

Not applicable.

**Supplementary Information:**

The online version contains supplementary material available at 10.1186/s12882-025-04442-3.

## Background

Acute interstitial nephritis (AIN) is a general term for diseases in which acute kidney injury (AKI) is accompanied by histological findings of interstitial inflammation, edema, and tubulitis [1]. While AIN can be caused by a variety of factors, including medications, autoimmune diseases, idiopathic forms, and infections (Legionella, Leptospira, Streptococcus, fungi, and viruses) [2], drug-induced AIN (DI-AIN) is the most common type, accounting for 71% of cases, compared with 15% for infection-related AIN, 8% for idiopathic AIN, 5% for tubulointerstitial nephritis with uveitis syndrome, and 1% for sarcoidosis [3]. In DI-AIN, medications trigger a T cell-mediated immune response that promotes tubulitis and interstitial inflammation with eosinophils, often resulting in enlarged, swollen kidneys that show increased echogenicity on ultrasound [1]. DI-AIN is observed in 2–3% of renal biopsy samples [4–6], with the percentage rising to 6.5–27% in patients with unexplained AKI [5–7], highlighting the importance of considering DI-AIN in AKI cases, particularly when nephromegaly is present.

Abemaciclib is a cyclin-dependent kinase (CDK) 4/6 inhibitor employed as an oral molecular-targeted drug for hormone receptor-positive, human epidermal growth factor receptor 2-negative metastatic breast cancers. It functions by blocking the G1-to-S phase transition in the cell cycle, preventing cell-cycle progression and cancer growth [8]. In clinical trials, the serum creatinine (Cr) level was demonstrated to increase by 15–40% after administration of abemaciclib, similar to the effects of two other CDK4/6 inhibitors, palbociclib and ribociclib [9]. These effects are thought to arise because CDK4/6 inhibitors and their main metabolites inhibit two organic cation transporters, multidrug and toxin extrusion (MATE) 1 and MATE2-K, consequently impairing Cr secretion by the renal tubules [10]; thus, the phenomenon is also known as pseudonephropathy. Although true AKI is a rare complication, six cases of acute renal failure following CDK4/6 inhibitor administration have been reported, comprising three cases with palbociclib, two cases with abemaciclib, and one case with ribociclib [11]. To date, there have been no reports of AIN associated with the administration of abemaciclib.

Here, we report a case of biopsy-proven AIN that developed shortly after the initiation of abemaciclib, leading to severe renal dysfunction with nephromegaly. Persistent renal dysfunction following fluid replacement for pre-renal AKI was successfully treated with glucocorticoid therapy.

## Case presentation

A 66-year-old Japanese woman visited a local doctor due to persistent diarrhea and nausea and was subsequently referred to our hospital with severe renal dysfunction (blood urea nitrogen, 128.7 mg/dL; Cr, 15.16 mg/dL). There was no clear evidence of chronic kidney disease prior to admission, and her serum Cr levels had been stable between 0.6 and 0.8 mg/dL. Approximately 11 months earlier, she had undergone a right nipple-sparing mastectomy with sentinel lymph node re-examination for right breast cancer (cT2N0M0; Stage IIA). Starting at 2 months post-surgery, she received dose-dense paclitaxel therapy for 5 months. At 2 weeks before her referral, abemaciclib was initiated as postoperative therapy but was subsequently discontinued within a few days due to diarrhea and vomiting. Despite treatment with loperamide and a probiotic drug containing *Clostridium butyricum*, her symptoms persisted, resulting in a weight loss of 2.1 kg and hypotension (blood pressure, 82/64 mmHg) noted by her local doctor. Her medical history included diabetes, and her regular medications comprised anastrozole, metformin, rosuvastatin, and vildagliptin. She was not taking any proton pump inhibitors, nonsteroidal anti-inflammatory drugs, antibiotics, or supplements.

**Fig. 1:**
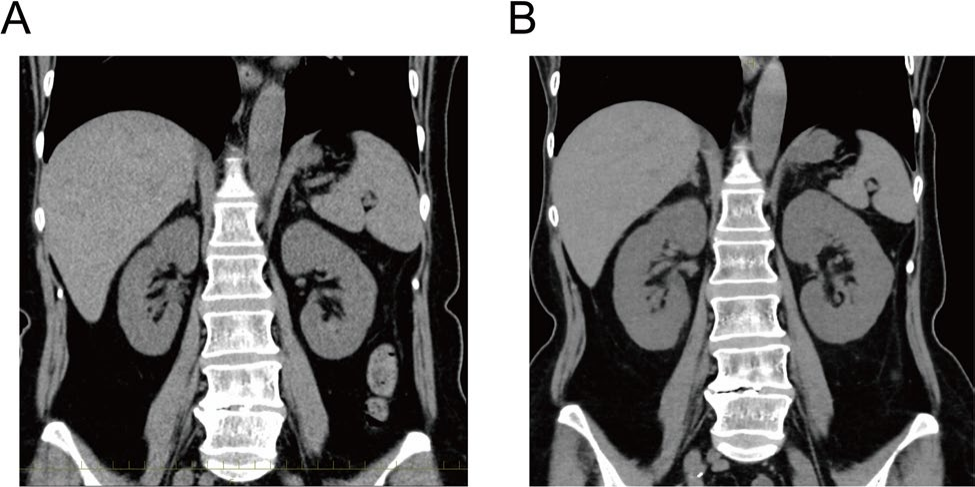
Computed tomography (CT) images of the abdomen. **A**, ** B** CT revealed bilateral nephromegaly at the diagnosis (B, right: 11.2 cm × 5.5 cm, left: 10.6 cm× 5.7 cm), compared to 6 years prior (A, right: 9.6 cm × 4.5 cm, left: 9.4 cm× 5.0 cm)

On admission, her blood pressure was 127/82 mmHg, and her heart rate was 90 beats per minute. A physical examination revealed no edema or signs of meningeal irritation. Laboratory data indicated renal failure with tubular injury (urinary β-2 microglobulin, 58.3 mg/L; N-acetyl-β-D-glucosaminidase, 24.8 IU/L) and metabolic acidosis (Table 1). Urinalysis and urine sediment examination revealed the presence of granular casts and epithelial cell casts, but there was no evidence of proteinuria, microscopic hematuria, or pyuria (Table 1). As pyuria was not observed, a urinary eosinophil examination was not performed. A urine culture was not carried out, because there were no clinical findings suggestive of a urinary tract infection: the patient had no fever, no pyuria, no nitrites in the urine, and only a minimal elevation of C-reactive protein. Serum protein electrophoresis revealed no M-protein peak; therefore, serum free light chain testing was not conducted. Fractional excretion of sodium (FENa) was elevated (11%), and autoantibodies were negative. A chest X-ray examination showed no cardiomegaly or pleural effusion, while a plain abdominal computed tomography examination revealed kidney enlargement (right: 11.2 cm × 5.5 cm; left: 10.6 cm × 5.7 cm) compared with the dimensions 6 years previously (right: 9.6 cm × 4.5 cm; left: 9.4 cm × 5.0 cm) (Fig. 1). Based on the elevated renal tubular damage markers, renal enlargement, and FENa value, intrinsic AKI was suspected, and the potentially nephrotoxic drugs were discontinued. Moreover, the clinical signs of volume depletion, including weight loss, inferior vena cava collapse (0–5 mm), hypotension, episodes of vomiting and poor oral intake, and pre-renal azotemia, indicated the presence of pre-renal AKI.

**Table 1:** Summary of the patient’s laboratory results

Parameter	Value	(Normal range)
(Urine)		
Urine specific gravity	1.005	
pH	7	
Urine protein/creatinine ratio (g/gCr)	0.31	(< 0.15)
Red blood cells (/HPF)	0–1	(< 5)
Granular casts (/WF)	1–4	Negative
Epithelial cell casts (/WF)	5–9	Negative
β2MG (mg/L)	58.3	(0.03–0.37)
NAG (IU/L)	24.8	(0–11.5)
(Blood)		
White blood cells (/µL)	3170	(3040–8540)
Red blood cells (104 /L)	387	(378–499)
Hemoglobin (g/dL)	11.1	(10.8–14.9)
Hematocrit (%)	31.8	(35.6–45.4)
Platelets (104 /L)	19.2	(15–36)
AST (U/L)	86	(13–33)
ALT (U/L)	34	(8–42)
LD (U/L)	418	(124–222)
Total protein (g/dL)	6.4	(6.7–8.3)
Serum albumin (g/dL)	3.9	(4–5)
Blood urea nitrogen (mg/dL)	124	(8–20)
Creatinine (mg/dL)	14.55	(0.4–0.7)
eGFR (mL/min/1.73m2)	2	(> 90)
Sodium (mmol/L)	129	(138–146)
Potassium (mmol/L)	5.6	(3.6–4.9)
Chloride (mmol/L)	85	(99–109)
Calcium (mg/dL)	8.7	(8.6–10.4)
Phosphorus (mg/dL)	9.8	(2.5–4.7)
Uric acid (mg/dL)	8.2	(2.3–7)
Plasma glucose (mg/dL)	163	(70–109)
Hemoglobin A1c (NGSP) (%)	5.6	(4.6–6.2)
C-reactive protein (mg/dL)	0.6	(< 0.2)
PT-INR	1.04	
APTT (sec)	29.1	(24–34)
Fib (mg/dL)	446.8	(200–400)
Immunoglobulin G (mg/dL)	867	(870–1700)
Immunoglobulin A (mg/dL)	91	(110–410)
Immunoglobulin M (mg/dL)	40	(46–260)
CH50 (U/mL)	51.1	(30–46)
C3 (mg/dL)	110	(86–160)
C4 (mg/dL)	43	(17–45)
Anti-nuclear antigen	Negative	Negative
Anti-neutrophilic cytoplasmic antibody	Negative	Negative
HBs-Ag	Negative	Negative
HCV-Ab	Negative	Negative

β2MG: β-2 microglobulin; NAG: N-acetyl-β-D-glucosaminidase

**Fig. 2:**
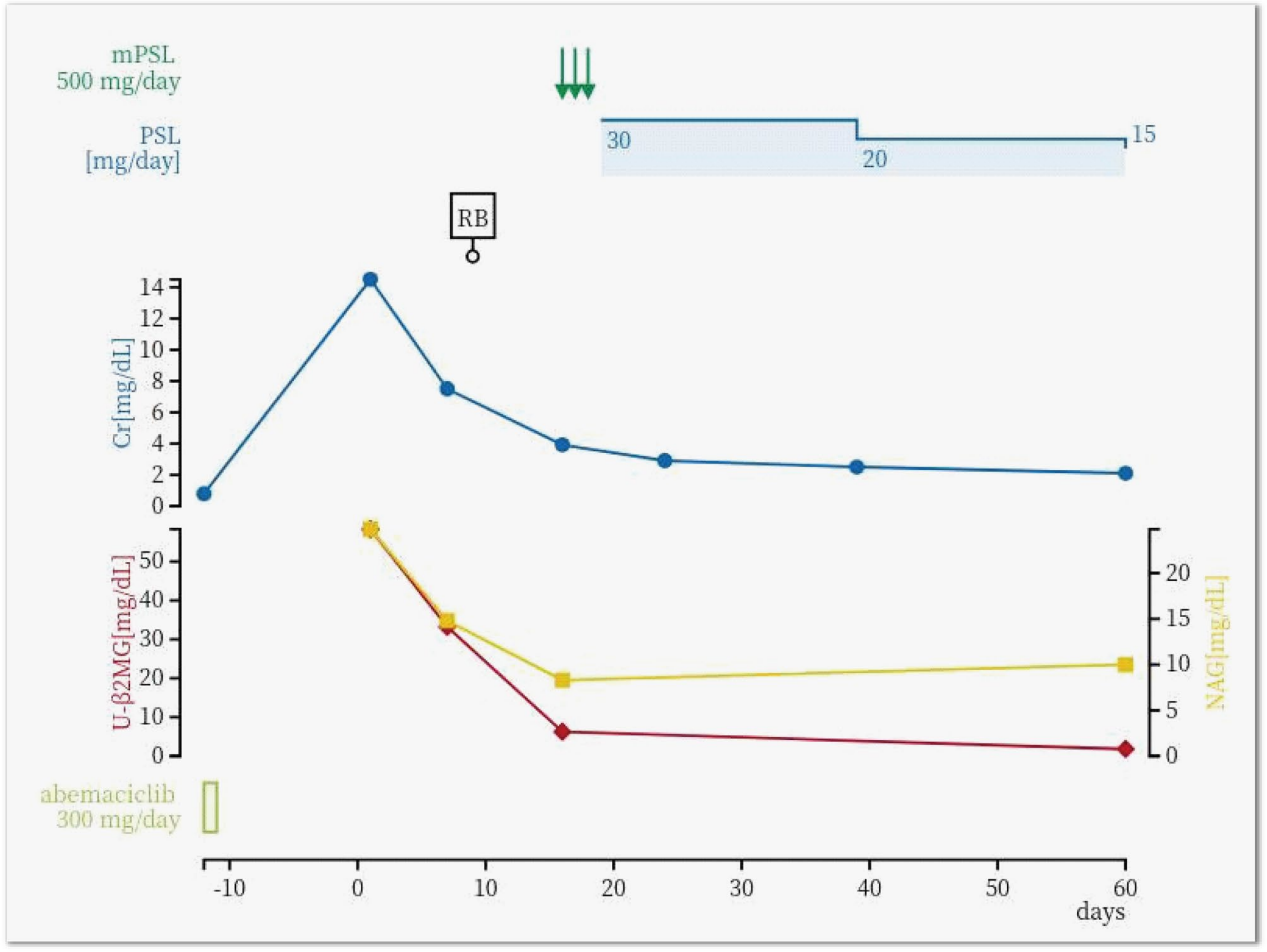
Time course of the patient’s renal function and tubular injury markers. Changes in serum creatinine (Cr, blue line), urinary β2-microglobulin (U-β2MG, red line), N-acetyl-β-D-glucosaminidase (NAG, yellow line). mPSL, methylprednisolone; PSL, prednisolone; RB, renal biopsy

**Fig. 3:**
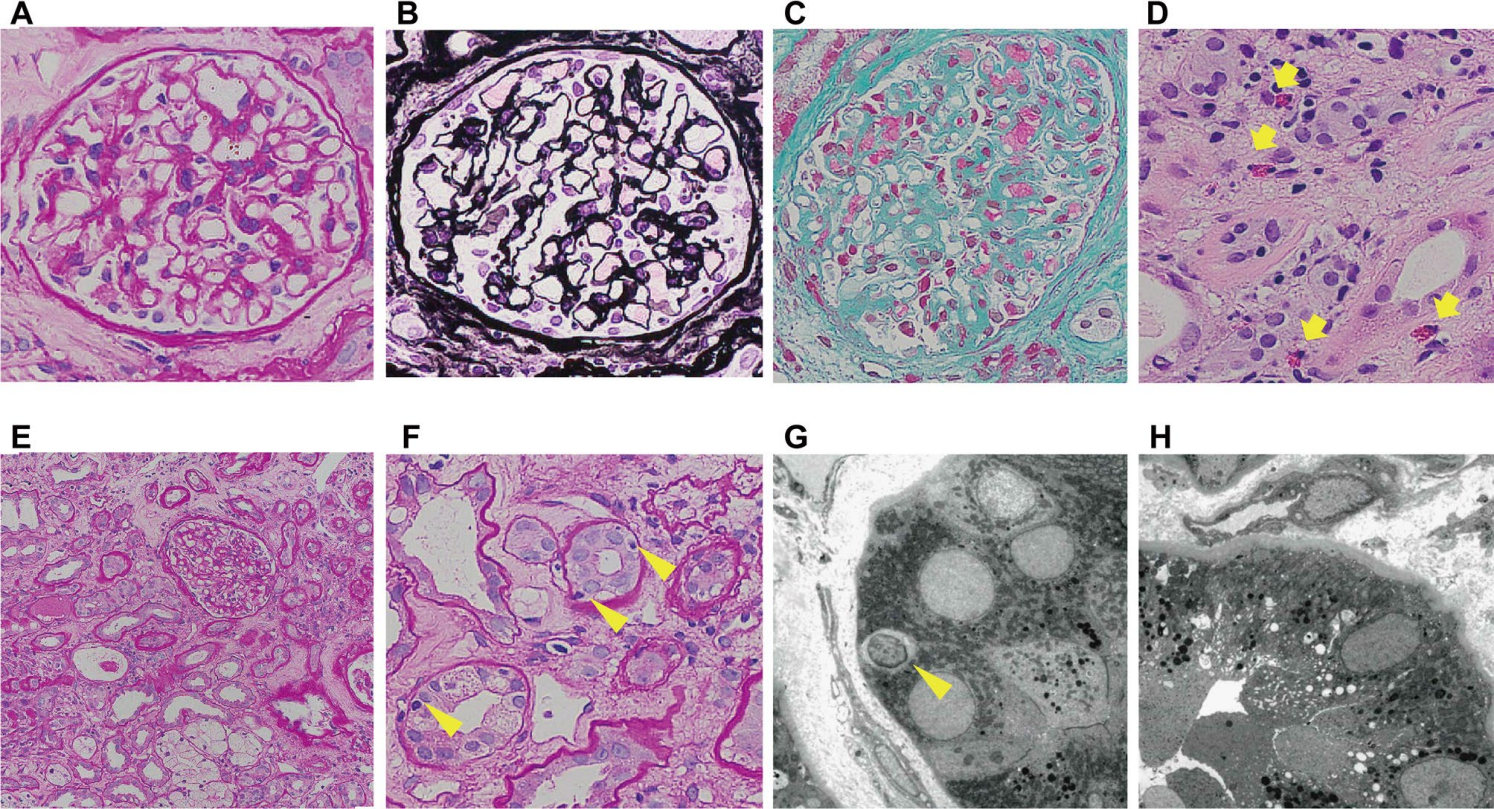
Light and electron microscopic views of the renal biopsy. **A–F** Light microscopic views of normal glomeruli (**A–C**) and interstitial infiltration of mononuclear cell with eosinophils (*indicated by yellow arrow*) (**D**,** E**) with tubulitis caused by mononuclear cells (*indicated by yellow arrowheads*) and damage to the epithelial cells of the renal tubules (**F**). Periodic acid–Schiff (**A**,** E**,** F**), Periodic acid–methenamine silver (**B**), Masson’s trichrome (**C**), hematoxylin-eosin staining (**D**) was used. **G**,** H** Electron microscopy confirmed tubulitis caused by mononuclear cells (*indicated by yellow arrowhead*) (**G**) and renal tubular damage with isometric vacuolization (**H**)

After fluid replacement was initiated, her renal function gradually improved, her vomiting resolved, and her weight returned to normal. However, the serum Cr level remained high (7.5 mg/dL on hospital day 7) (Fig. 2). To investigate the cause of the renal failure and kidney enlargement, a renal biopsy was performed on day 9. A total of 25 glomeruli without segmental or global sclerosis were obtained. Light microscopic examination revealed normal glomeruli but showed interstitial infiltration of mononuclear cells and eosinophils, along with tubulitis caused by mononuclear cells and proximal tubular damage with isometric vacuolization, suggesting interstitial nephritis and tubular necrosis (Fig. 3). Thickening of the arteriole and interlobular artery walls was observed, which was presumed to be related to the underlying diabetes and dyslipidemia. Masson’s trichrome staining revealed fibrosis, which affected approximately 30% of the tissue sample. Although some vascular changes were observed, glomerular involvement was minimal, with no nodular or diffuse lesions, no globally sclerotic glomeruli, and no arteriolar hyalinization. Therefore, the observed tubulointerstitial fibrosis and areas of isometric vacuolization were considered largely attributable to AIN and proximal tubular injury rather than to diabetic pathology. Immunofluorescence analysis showed no significant deposition of immunoglobulin and complement, while electron microscopy confirmed inflammatory cell infiltration in the renal tubules and damage to renal tubular epithelial cells. Given that abemaciclib was the only drug initiated just before the onset of AKI, we considered that this drug was the most likely cause of the AIN. Methylprednisolone 500 mg/day was administered from days 16 to 18, followed by prednisolone (PSL) 30 mg/day starting on day 19. The Cr levels and renal tubular damage markers gradually improved, with the Cr level decreasing to approximately 2 mg/dL within 2 months (Fig. 2).

### Discussion and conclusions

We report a case of nonoliguric AKI with bilateral nephromegaly that developed shortly after the initiation of abemaciclib for breast cancer, in which both AIN and acute tubular damage were confirmed by a renal biopsy. AKI is not a well-described complication of CDK4/6 inhibitor administration in pharmacological studies, although an increase in the serum Cr level without true renal injury has been noted [12]. To date, only six cases of AKI associated with CKD4/6 inhibitor administration have been reported, with a renal biopsy performed in all six cases (Table 2) [11]. In five cases, AKI resulted from acute tubular necrosis after prolonged treatment (median onset: 278 days) (Table 2). Only one case receiving palbociclib exhibited interstitial nephritis with proteinuria, hematuria, and pyuria, but the time to AIN was not specified (Table 2). In contrast, the present case developed AIN within 2 weeks of abemaciclib initiation, without abnormal urinalysis findings. DI-AIN typically occurs at 7–10 days after drug exposure [1, 13], and a patient may have few or no clinical symptoms or signs of the renal disorder [1]. These findings suggest that while CDK4/6 inhibitor-induced AIN is rare, it can occur after short-term treatment without clinical symptoms, similar to AIN induced by other drugs.

**Table 2:** Clinical features of CDK4/6 inhibitor-associated AKI

Pt [ref]	Age/Sex	Mallgnacy	Cancer Stage	CDK4/6 Type	Baseline SCr (mg/dL)	Peak SCr (mg/dL)	Time to AKI (days)	Urinalysis	Renal pathology
1 [11]	76/F	Breast	IV	Palbociclib	NA	7.0	NA	protein, blood, 100 WBCs	AIN
2 [11]	81/F	Breast	IV	Palbociclib	1.2	4.0	NA	LE, protein	ATN
3 [11]	83/F	Breast	IV	Palbociclib	1.3	2.2	210	protein, blood	ATN
4 [11]	57/F	Breast	IV	Abemaciclib	0.9	5.9	346	NA	ATN
5 [11]	77/F	Breast	IV	Ribociclib	0.8	11.2	683	protein, blood, granular casts	ATN
6 [11]	58/F	Brain	IV	Abemaciclib	1.0	1.9	102	protein, blood, muddy brown casts	ATN
Present case	66/F	Breast	II	Abemaciclib	0.7	14.6	13	−	AIN

AKI: acute kidney injury; LE: leukocyte esterase; NA: not available; Pt: patient; SCr: serum creatinine; WBCs: white blood cells

To the best of our knowledge, this is the first reported case of AIN associated with the administration of abemaciclib. In this case, glucocorticoid therapy led to improved renal function, with a reduction in renal tubular damage markers. In previous cases of AIN associated with the use of CDK4/6 inhibitors, glucocorticoid therapy was not administered because the patients were transitioned to comfort care and died (Table 2). In contrast, our patient had no postoperative recurrence and remained in good general condition, allowing glucocorticoid therapy to be initiated. Since benefits of glucocorticoid therapy were observed in the present case, this treatment option should be considered for cases of CDK4/6 inhibitor-induced AIN, similar to other forms of DI-AIN, particularly when patients are in good general condition and their cancer is well controlled.

As the present patient developed severe AKI, and a renal biopsy revealed eosinophil infiltration and acute tubulitis, steroid pulse therapy was administered, followed by PSL 30 mg/day. Typically, in patients with DI-AIN, high-dose steroids are administered for 2–3 weeks, followed by a taper over 6–8 weeks [14]. While no studies have shown a clear advantage of intravenous (IV) steroids over high-dose oral steroids, IV pulse steroids may be considered for severe AKI [15]. Meanwhile, the timing of glucocorticoid administration may be a critical factor in DI-AIN [16]. In a multicenter retrospective study involving 61 cases of biopsy-proven DI-AIN, a delay in steroid initiation (34 days versus 13 days) was associated with poor renal recovery [17]. However, another study found that while the steroid-treated group showed greater improvement in the serum Cr level, there was no clear correlation between eGFR improvement and the timing of steroid initiation [18]. Therefore, although early steroid administration is desirable, this therapy should still be considered in cases where the diagnosis is challenging and delayed. In our case, steroid treatment was initiated on day 16 after confirming the renal biopsy results, because CDK4/6 inhibitors can cause both AIN and ATN, the latter of which has no specific treatment and is managed by supportive care. Since differentiation between these two conditions is difficult using laboratory data alone, an early renal biopsy plays a crucial role in assessing the disease activity and determining the treatment strategies in patients with CDK4/6 inhibitor-induced AKI.

Another notable feature of the present case is the complication of pre-renal AKI. The patient had the highest peak Cr level among the reported cases of CDK4/6 inhibitor-associated AKI (Table 2), but did not require renal replacement therapy (RRT) because her uremic symptoms, including vomiting, were resolved by fluid replacement. In contrast, a previously reported patient with ribociclib-associated AKI had a lower peak Cr level (11.2 mg/dL) but required RRT for 6 months following the AKI event [11]. One possible explanation for this difference may be the higher incidence of diarrhea as a side effect of abemaciclib compared with ribociclib. Although gastrointestinal toxicities, such as anorexia, nausea, and diarrhea, are common to all CDK4/6 inhibitors, clinical trials have indicated that diarrhea occurred in 81% of patients taking abemaciclib, compared with 35% of patients taking ribociclib [19, 20]. Abemaciclib-induced diarrhea typically appears within the first few days of treatment and can sometimes be severe, requiring dose adjustments or supportive treatment. In our case, the patient experienced persistent diarrhea for at least 1 week after starting abemaciclib, leading to a weight loss of 2.1 kg. These observations highlight the importance of considering pre-renal AKI as a potential complication for this side effect, particularly when severe renal dysfunction is observed shortly after abemaciclib administration compared with administration of other CDK4/6 inhibitors.

In this case, pre-renal AKI was initially suspected based on the physical findings and clinical course. However, her renal enlargement, elevated tubular injury markers, and poor response to fluid resuscitation raised suspicion for intrinsic AKI, leading to a renal biopsy. In cases with onconephrology, clinicians should consider intrinsic causes when the renal dysfunction appears disproportionate to the pre-renal factors. Recognition of red flags, such as persistent renal dysfunction, abnormal urinary findings, or renal enlargement, may help to avoid anchoring bias and facilitate timely diagnosis.

CDK4/6 inhibitors can cause pseudo-AKI by impairing the tubular secretion of Cr. Therefore, measurement of the cystatin C level is often useful for distinguishing pseudo-AKI from true AKI. In this case, the cystatin C level on admission was 4.53 mg/L, supporting the presence of true AKI rather than pseudo-AKI. Since cystatin C levels typically plateau at approximately 5 mg/L [21], we used eGFR based on serum Cr in this case. However, measurement of cystatin C is considered important for differentiating pseudo-AKI from true AKI, even when the cystatin C level is near the plateau.

In conclusion, we report a case of biopsy-proven AIN that developed shortly after the initiation of abemaciclib for breast cancer, leading to severe renal dysfunction with bilateral nephromegaly. The renal function improved with glucocorticoid therapy following fluid replacement for pre-renal AKI. Although both AIN and ATN can be caused by prolonged use of CDK4/6 inhibitors, AIN can also occur after short-term use. An early renal biopsy is crucial for differentiating AIN from ATN and determining the need for glucocorticoid therapy.

## Supplementary Information

Below is the link to the electronic supplementary material.

Supplementary Material 1

## Data Availability

The datasets used in this case report are available from the corresponding author on reasonable request.

## Abbreviations

AIN: Acute interstitial nephritis
AKI: Acute kidney injury
ATN: Acute tubular necrosis
β2MG: β-2 microglobulin
Cr: Creatinine
CDK: Cyclin-dependent kinase
DI-AIN: Drug-induced acute interstitial nephritis
FENa: Fractional excretion of sodium
IV: Intravenous
MATE: Multidrug and toxin extrusion
NAG: N-acetyl-β-D-glucosaminidase
PSL: Prednisolone
RRT: Renal replacement therapy

## References

[1] Perazella MA, Markowitz GS. Drug-induced acute interstitial nephritis. Nat Rev Nephrol. 2010;6:461–70. 20517290 10.1038/nrneph.2010.71

[2] Yang C-W, Wu M-S, Pan M-J, Hsieh W-J, Vandewalle A, Huang C-C. The leptospira outer membrane protein LipL32 induces tubulointerstitial Nephritis-Mediated gene expression in mouse proximal tubule cells. J Am Soc Nephrol. 2002;13:2037–45. 12138134 10.1097/01.asn.0000022007.91733.62

[3] Richard J, Baker CD, Pusey. The changing profile of acute tubulointerstitial nephritis. Nephrol Dial Transplant. 2004;19:8–11. 10.1093/ndt/gfg464 14671029

[4] Buysen JGM, Houthoff HJ, Krediet RT, Arisz L. Acute interstitial nephritis: a clinical and morphological study in 27 patients. Nephrol Dialysis Transplantation. 1990;5:94–9. 10.1093/ndt/5.2.94 2113219

[5] Davison AM, Jones CH. Acute interstitial nephritis in the elderly: a report from the UK MRC glomerulonephritis register and a review of the literature. Nephrol Dial Transplant. 1998;13 suppl_7:12–6. 10.1093/ndt/13.suppl_7.12 9870431

[6] Clarkson MR, Giblin L, O’Connell FP, O’Kelly P, Walshe JJ, Conlon P, et al. Acute interstitial nephritis: clinical features and response to corticosteroid therapy. Nephrol Dial Transplant. 2004;19:2778–83. 10.1093/ndt/gfh485 15340098

[7] Farrington K, Levison DA, Greenwood RN, Cattell WR, Baker LR. Renal biopsy in patients with unexplained renal impairment and normal kidney size. Int J Med. 1989;70:221–33. 2602535

[8] Finn RS, Aleshin A, Slamon DJ. Targeting the cyclin-dependent kinases (CDK) 4/6 in Estrogen receptor-positive breast cancers. Breast Cancer Res. 2016;18:17. 26857361 10.1186/s13058-015-0661-5 PMC4746893

[9] Patnaik A, Rosen LS, Tolaney SM, Tolcher AW, Goldman JW, Gandhi L, et al. Efficacy and safety of abemaciclib, an inhibitor of CDK4 and CDK6, for patients with breast cancer, non–small cell lung cancer, and other solid tumors. Cancer Discov. 2016;6:740–53. 27217383 10.1158/2159-8290.CD-16-0095

[10] Sledge GW, Toi M, Neven P, Sohn J, Inoue K, Pivot X, et al. The effect of abemaciclib plus fulvestrant on overall survival in hormone receptor-positive, ERBB2-negative breast cancer that progressed on endocrine therapy - MONARCH 2: a randomized clinical trial. JAMA Oncol. 2020;6:116–24. 31563959 10.1001/jamaoncol.2019.4782 PMC6777264

[11] Gupta S, Caza T, Herrmann SM, Sakhiya VC, Jhaveri KD. Clinicopathologic features of acute kidney injury associated with CDK4/6 inhibitors. Kidney Int Rep. 2022;7:618–23. 35257075 10.1016/j.ekir.2021.11.033 PMC8897293

[12] Chappell JC, Turner PK, Pak YA, Bacon J, Chiang AY, Royalty J, et al. Abemaciclib inhibits renal tubular secretion without changing glomerular filtration rate. Clin Pharmacol Ther. 2019;105:1187–95. 30449032 10.1002/cpt.1296 PMC6465099

[13] Rossert J. Drug-induced acute interstitial nephritis. Kidney international. Blackwell Publishing Inc.; 2001;60:804–17. 10.1046/j.1523-1755.2001.060002804.x 11473672

[14] Donati A, Krishnan N. Should corticosteroids be used to treat biopsy-proven drug-induced acute interstitial nephritis: PRO. Kidney360. 2022;3:1306–9. 10.34067/KID.0006642021 PMC9416830 36176666

[15] Abdul Majeed Chowdry. Drug-induced acute interstitial nephritis: prospective randomized trial comparing oral steroids and high-dose intravenous pulse steroid therapy in guiding the treatment of this condition. Saudi J Kidney Dis Transpl. 2018;29:598–607. 29970736 10.4103/1319-2442.235171

[16] Perazella MA, Rosner MH. Drug-Induced acute kidney injury. Clin J Am Soc Nephrol. 2022;17:1220–33. 35273009 10.2215/CJN.11290821 PMC9435983

[17] González E, Gutiérrez E, Galeano C, Chevia C, De Sequera P, Bernis C, et al. Early steroid treatment improves the recovery of renal function in patients with drug-induced acute interstitial nephritis. Kidney Int. 2008;73:940–6. 18185501 10.1038/sj.ki.5002776

[18] Muhammad N, Raza M, Hadid CE, Keen C, Bingham AHJ, Salmon. Acute tubulointerstitial nephritis, treatment with steroid and impact on renal outcomes. Nephrology. 2012;17:748–53. 22817666 10.1111/j.1440-1797.2012.01648.x

[19] Matthew P, Goetz M, Toi M, Campone J, Sohn. MONARCH 3: abemaciclib as initial therapy for advanced breast cancer. J Clin Oncol. 2017;35:3638–46. 28968163 10.1200/JCO.2017.75.6155

[20] Hortobagyi GN, Stemmer SM, Burris HA, Yap YS, Sonke GS, Paluch-Shimon S, et al. Updated results from MONALEESA-2, a phase III trial of first-line ribociclib plus letrozole versus placebo plus letrozole in hormone receptor-positive, HER2-negative advanced breast cancer. Ann Oncol. 2018;29:1541–7. 29718092 10.1093/annonc/mdy155

[21] Horio M, Enyu Imai Y, Yasuda T, Watanabe, Seiichi Matsuo. Performance of serum cystatin C versus serum creatinine as a marker of glomerular filtration rate as measured by inulin renal clearance. Clin Exp Nephrol. 2011;15:868–76. 21861242 10.1007/s10157-011-0525-y

